# Membrane Active Peptides and Their Biophysical Characterization

**DOI:** 10.3390/biom8030077

**Published:** 2018-08-22

**Authors:** Fatma Gizem Avci, Berna Sariyar Akbulut, Elif Ozkirimli

**Affiliations:** 1 Bioengineering Department, Marmara University, Kadikoy, 34722 Istanbul, Turkey; gizemavci@gmail.com (F.G.A.); berna.akbulut@marmara.edu.tr (B.S.A.); 2 Chemical Engineering Department, Bogazici University, Bebek, 34342 Istanbul, Turkey

**Keywords:** antimicrobial peptides, cell-penetrating peptides, biophysical characterization, uptake mechanism, membrane disruption, peptide–lipid interactions

## Abstract

In the last 20 years, an increasing number of studies have been reported on membrane active peptides. These peptides exert their biological activity by interacting with the cell membrane, either to disrupt it and lead to cell lysis or to translocate through it to deliver cargos into the cell and reach their target. Membrane active peptides are attractive alternatives to currently used pharmaceuticals and the number of antimicrobial peptides (AMPs) and peptides designed for drug and gene delivery in the drug pipeline is increasing. Here, we focus on two most prominent classes of membrane active peptides; AMPs and cell-penetrating peptides (CPPs). Antimicrobial peptides are a group of membrane active peptides that disrupt the membrane integrity or inhibit the cellular functions of bacteria, virus, and fungi. Cell penetrating peptides are another group of membrane active peptides that mainly function as cargo-carriers even though they may also show antimicrobial activity. Biophysical techniques shed light on peptide–membrane interactions at higher resolution due to the advances in optics, image processing, and computational resources. Structural investigation of membrane active peptides in the presence of the membrane provides important clues on the effect of the membrane environment on peptide conformations. Live imaging techniques allow examination of peptide action at a single cell or single molecule level. In addition to these experimental biophysical techniques, molecular dynamics simulations provide clues on the peptide–lipid interactions and dynamics of the cell entry process at atomic detail. In this review, we summarize the recent advances in experimental and computational investigation of membrane active peptides with particular emphasis on two amphipathic membrane active peptides, the AMP melittin and the CPP pVEC.

## 1. Introduction

Peptides that interact with the cell membrane by disrupting it, by passing through it, or by residing at the membrane interface and fusing with it are known as membrane active peptides. There are two major classes of membrane active peptides; antimicrobial peptides (AMPs), that kill cells, and cell-penetrating peptides (CPPs), that carry cargos across lipid bilayers [1].

**Figure 1:**
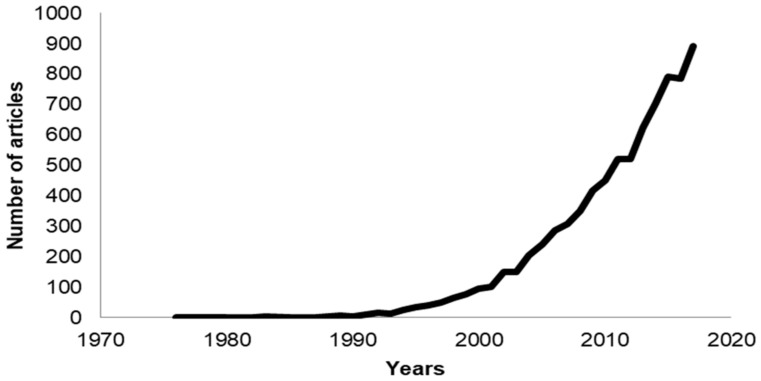
Number of publications in the past 40 years that use the phrases antimicrobial peptide, cell-penetrating peptide, or membrane active peptide.

There has been an exponential increase in the number of publications that have the keywords “antimicrobial peptide”, “cell-penetrating peptide”, or “membrane active peptide”. The field of AMPs is especially more intense due to the urgent need for novel antimicrobials, with more than 600 articles published in 2017 (Figure 1). In contrast to antimicrobials that target proteins, cells cannot readily build resistance to AMPs that target the cell membrane because it is difficult, if not impossible, to alter this crucial cell structure [2]. Antimicrobial peptides have been shown to have antiviral [3] and anticancer [4] activities as well. Currently (May 2018), there are 2981 AMPs curated by the APD3 database [5], in which more than 1000 of them are peptides from amphibians and about 200 are human host defense peptides. There are 1855 CPPs reported in the CPPsite 2.0 database [6].

In this review, we summarize some of the most recent work on the structural elucidation of AMPs and CPPs and their action mechanisms. We first introduce examples of AMPs and CPPs and provide a general overview of the currently proposed models for their membrane activity. Then, we introduce some of the advanced techniques that provide temporal and spatial resolution about peptide–membrane interactions and then review the recent advances in the deciphering of AMP and CPP mechanism. Finally, we focus on two amphipathic peptides, namely melittin and pVEC. Melittin is an AMP derived from bee venom and pVEC is a CPP derived from murine vascular endothelial cadherin protein. We provide an outlook based on elucidation of membrane action toward the design of novel peptide-based drugs.

## 2. Antimicrobial Peptides

Antimicrobial peptides, also known as host defense peptides (HDPs), kill bacteria, virus, and fungi either by disrupting their membrane integrity or by inhibiting some cellular functions [7]. The discovery of the first AMP, gramicidin, in 1939 [8] opened up the field on AMPs, which have gained continued interest as a result of the focus on the discovery of novel antimicrobials due to increasing microbial resistance to conventional antibiotics. Their unique mode of action together with their multitarget properties have made AMPs promising candidates for the development of new drug leads. Antimicrobial peptides are part of the host defense system of different organisms [9] including humans (defensin [10], dermcidin [11], and LL-37, also known as cathelicidin [12]), plants (defensin [13,14,15]), bees (melittin [16]), insects (cecropin [17]), and amphibians (magainin [18]).

Antimicrobial peptides generally consist of fewer than 100 amino acid residues [19]. These are usually L-amino acids, but AMPs may also contain modified residues such as disulfide linkages or lanthionines [20,21]. Commonly, they have a positive net charge (from +4 to +6) usually due to a stretch of arginine and/or lysine residues that interact with the negatively charged phosphate head groups of the bacterial membrane. They also contain a hydrophobic region, making them amphipathic and membrane active. However, bacteria have gained resistance against cationic AMPs by acquiring positively charged groups on their outer cell wall and to combat these bacteria some anionic AMPs have also evolved, one of which is dermcidin found in human sweat [11,22,23]. There are also AMPs with a net negative charge (from −1 to −7), such as chromacin [24], but they are believed to have other primary biological roles [19].

**Figure 2:**
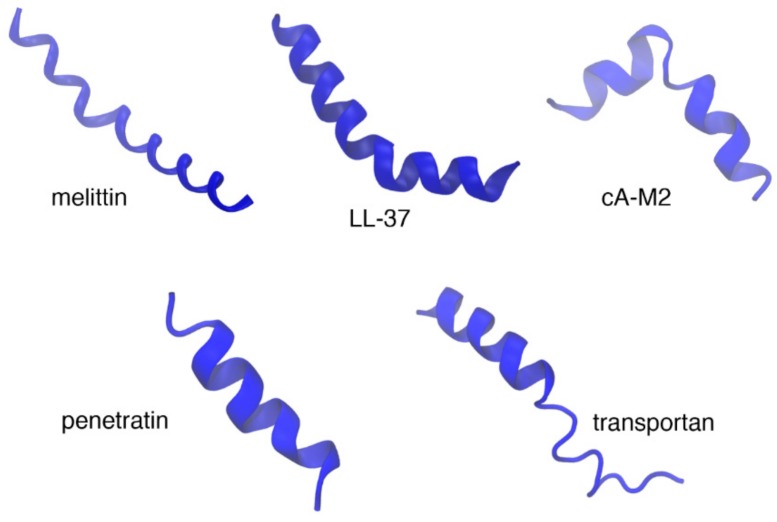
Examples of α-helical antimicrobial peptides (AMPs) (top panel) and cell-penetrating peptides (CPPs) (bottom panel). Top panel shows the X-ray crystal structure of melittin (2mlt), solution structure of LL-37 (cathelicidin) in deuterated sodium dodecyl sulfate (dSDS) micelles (2k6o) [34], and the solution structure cecropin A(1-8)–magainin 2(1-12) hybrid peptides (1d9j) [35]. The solution structures of CPPs penetratin in negatively charged phospholipid bicelles (1omq) [36] and transportan in neutral phospholipid bicelles (1smz) [37] are shown in the bottom panel.

**Figure 3:**
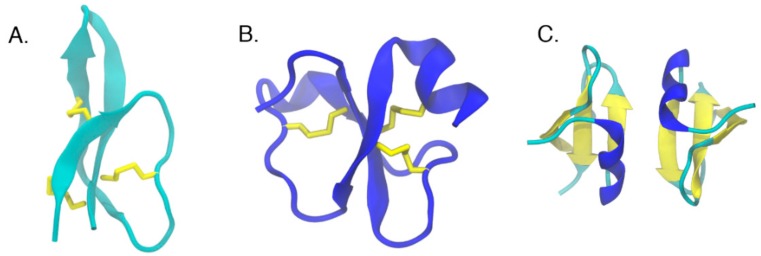
Examples of cyclic peptides with a β-sheet core. (**A**) Solution structure of human defensin 5 (2lxz) [27], (**B**) crystal structures of crotamine (4gv5) [38], (**C**) and human β-defensin-2 (1fd3) [20]. Disulfide bridges holding the β-strands together are shown in yellow stick representation.

The secondary structure of AMPs is diverse. Clustering of 135 nuclear magnetic resonance (NMR) structures of AMPs based on backbone dihedral angles shows that they can assume a wide variety of secondary structures ranging from completely helical to all beta [25]. Antimicrobial peptides can be classified into four subgroups based on their secondary structure; the first group includes linear, α-helical peptides (melittin, dermcidin, and LL-37) (Figure 2) [12,22,26], the second group includes peptides with β-strands connected by two or more disulfide bridges (defensins and protegrins) (Figure 3) [20,27], the third, AMPs with intermolecular disulfide bonds exhibiting loop/hairpin-like structures, (such as bactenecin), and the final group are peptides with special or modified amino acids (such as the proline/arginine-rich peptide Bac7 or the lanthionine containing lantibiotics) [28,29,30,31]. Even in the membrane bound state, the peptide can assume various different conformations depending on the concentration [32]. Another group of AMPs, such as indolicidin from the cathelicidin family [33], do not have a well-defined secondary structure. Several researchers have proposed that a general mechanism of antimicrobial action may not exist because of the diversity of AMP structures.

The initial contact of most AMPs with the microbial membranes is commonly achieved nonspecifically though electrostatic and hydrophobic interactions. Following this initial contact, different models try to explain the action of AMPs. The mechanism of action of AMPs can generally be categorized under pore-forming and nonpore-forming models [39].

The generally accepted pore-forming models are known as barrel-stave and toroidal pore-forming models. The barrel stave pores use the bilayer hydrocarbon core as a template for peptide-self-assembly, thus the lipid bilayer is barely perturbed [40]. The peptides are oriented perpendicular to the plane of the membrane to form a fairly rigid cylindrical barrel. In this model, initially AMPs bind parallel to the membrane surface as a monomer, followed by oligomerization and pore formation. As they insert into the membrane, the peptides usually assume an amphipathic secondary structure in which the hydrophobic regions interact with the membrane lipids, while the hydrophilic regions form the lumen of the channel [31,41]. They could be α-helical or β-sheet structures but their minimum length should span the lipid bilayer [39]. Alamethicin [42] and pardaxin [43,44] are among these peptides that make barrel stave pores.

In the toroidal pore model, peptides are known to disrupt the normal segregation of polar and nonpolar parts of the membrane [40]. As peptides are inserted into the membrane, they form a bundle [45,46] and induce the lipid monolayers to bend [31]. The lipid structure around a toroidal pore is strongly affected as some of the lipids participate directly in the pore, in contact with the peptides. In this toroidal structure, the peptides can be either perpendicular or tilted with respect to the plane of the membrane [41]. Such pores are transient allowing the peptide to enter the cytoplasm and target intracellular components [47]. The ion selectivities and discrete sizes are other features of toroidal pores [48]. Variants of the classical toroidal pore, such as huge toroidal pore (for lacticin Q) and disordered toroidal pore (for melittin) have also been described [49]. The term ‘disordered toroidal’ pore was introduced to describe pores that are toroidally shaped but only one or two peptides line the pore with partial participation by the peptides in the pore. ‘Huge toroidal’ pores only form above a critical peptide to lipid ratio and require local aggregation of peptides [41,50]. Melittin [51] and magainins [52] are examples of toroidal pore-forming peptides.

Antimicrobial peptides do not necessarily act by pore formation; they may induce their effects through nonspecific membrane permeabilization. One of the most common nonpore-forming models is known as the carpet model [19,49,53]. In this model, AMPs are adsorbed on the membrane surface in a detergent-like manner and therefore affect the membrane architecture. The interactions are first driven by electrostatics and when a threshold concentration of the AMPs on the membrane surface is reached, peptides cover the surface of the membrane in a carpet-like manner. At this stage the membrane structure is destabilized and no longer can be maintained, thus it disintegrates [31,49]. Antimicrobial peptides such as aurein 1.2 [54] and cecropin c1 [55] impose their activities using this model.

**Figure 4:**
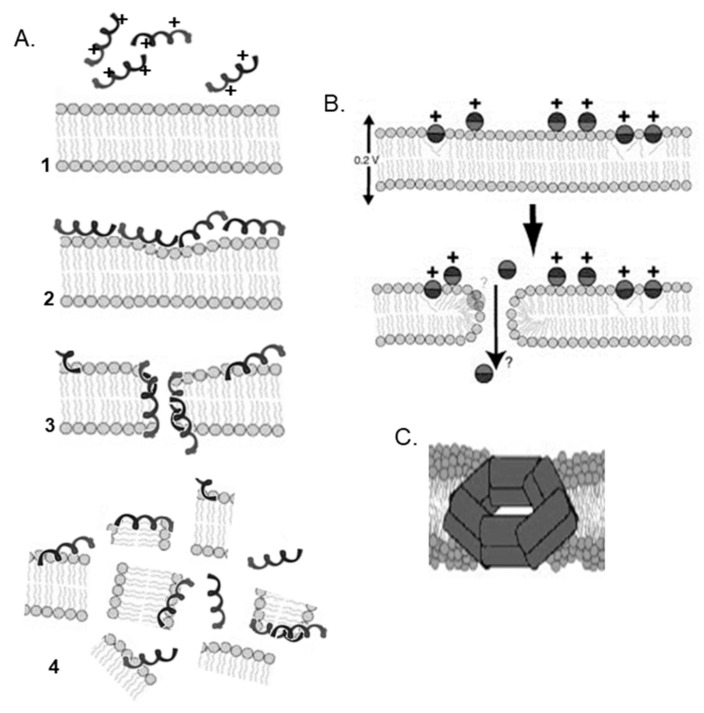
Different pore models. (**A**) Shai–Huang–Matsazuki (SHM) model [9]. (**B**) Electroporation model (reprinted from a previous paper [33], Copyright (2006), with permission from Elsevier). (**C**) Double-belt pore model (reprinted with permission from a previous paper [41]. Copyright (2014) American Chemical Society).

In addition to the aforementioned three models, other models have also been proposed, such as the Shai–Huang–Matsazuki (SHM) model, electroporation model, amyloid formation model, and double-belt pore model (Figure 4). See Lee et al. for a brief list of the different mechanisms used by AMPs [49].

The stability of the pores has been a question of interest. Marquette and Bechinger have proposed the so-called ‘soft membrane adapt and respond, also transiently’ (SMART) model based on microscopic imaging [56]. In this model, the peptide induces transient pores at low concentrations and membrane disintegration at high concentrations.

Initially AMPs were recognized by their ability to interact strongly with cell membranes and disrupt their integrity. Later studies have demonstrated their immunomodulatory and chemotactic properties [57]. Some AMPs can interact with intracellular targets to induce cell damage and eventually cell death by inhibition of cell wall, DNA, RNA, or protein synthesis [7,31,58]. Nisin inhibits cell wall synthesis [59], while drosocin, pyrrhocoricin, and apidaecin interact with DNA and RNA [60,61]. Human cathelicidin LL-37, which is produced by neutrophils, has not only antimicrobial activity but it also regulates inflammatory response and it is involved in carcinogenesis [62]. LL-37 also has chemotactic properties, mediated through membrane receptors [63]. Similarly, defensins produced mainly by lymphocytes have been shown to participate in innate as well as adaptive immunity [64]. 

The diverse activities of AMPs may depend on peptide concentration, cell type, and membrane properties, and elucidation of the mechanism of action of these AMPs is the key in understanding the conditions in which these peptides function. 

## 3. Cell-Penetrating Peptides

Cell-penetrating peptides, also referred to as protein-transduction domains (PTDs), are a diverse set of membrane active peptides of fewer than 30 residues, commonly with a net positive charge. They are known to facilitate the delivery of various biomolecules across cellular membranes of eukaryotic cells with limited toxicity. The molecular weight of the bioactive cargo, which may be linked covalently or noncovalently, may be several times greater than the molecular weight of the CPP [65,66]. Among their cargos, are plasmid DNA, oligonucleotides, siRNA (short interfering RNA), PNA (peptide nucleic acid), proteins and peptides, imaging agents (fluorescent dyes and quantum dots), drugs, as well as liposome nanoparticles [67,68,69,70,71].

The cationic Tat was the first CPP discovered [72] followed by penetratin [36,73]. Tat fused to the 120 kDa β-galactosidase penetrated through the blood brain barrier in a nontoxic fashion and distributed throughout the brain [74]. Penetratin, derived from the third helix of the antennapedia protein homeodomain from Drosophila, could directly penetrate into giant unilamellar vesicles (GUVs) [75,76]. The amphipathic peptides MPG and Pep-1 (also known as Chariot) consist of three domains: a hydrophobic motif at their N-terminal, a hydrophilic, lysine-rich domain, and a linker domain (WSQP) that enhances the flexibility of the hydrophobic and hydrophilic domains [67]. Another amphipathic peptide, CADY, combines aromatic tryptophan and cationic arginine residues into a self-assembling peptide [77]. Pep-1 could deliver full length antibodies and proteins as big as 120 kDa with an efficiency greater than 80% [78]. Interaction of the amphipathic peptide, specifically tryptophan residues in the hydrophobic domain, with peptidoglycans are critical in the eventual internalization process [79]. Among the oldest hydrophobic peptides is transportan 10 (TP10), derived from transportan which is a chimeric peptide of the neuropeptide galanin and wasp venom mastoparan linked by a lysine chain [32,80]. It was reported to sink deeply into the bilayer and cross it carrying its cargo such as green fluorescent protein (GFP) [81].

Cell penetrating peptides have been curated [82] in the CPP site database that contains information on 843 CPPs. Analysis of these peptides for amino acid composition shows that arginine (Arg), lysine (Lys), leucine (Leu), and alanine (Ala) are more abundant, with the cationic residues showing higher preference than other proteins in the SwissProt [83]. However, CPPs are variable in terms of amino acid composition and three-dimensional (3D) structure, with examples of cationic, anionic, and neutral sequences having different degrees of hydrophobicity and polarity. In general, CPPs do have sequence homology, which leads to different modes and different levels of uptake [84]. The number of CPPs with structural information is limited. Most of them assume a random coil conformation [85]. An increase in helicity often improves penetration. For those with secondary structure assignments, only a few have their 3D structures determined. The solution structures of penetratin in model systems such as sodium dodecyl sulfate (SDS) micelles and dimyristoylphosphatidylcholine/dimyristoylphosphatidylglycerol/dihexanoylphosphatidylcholine (DMPC/DMPG/DHPC) bicelles [86], transportan in bicelles with a fraction of the negatively charged DMPG incorporated in the bilayer [87] or neutral phospholipid bicelles [37] are a few representative examples (Figure 2). The classification of CPPs may be based on similarities in origin, sequence, chemical charge, binding type, and general physicochemical properties [88].

Based on their origin, CPPs can be separated into natural and artificial peptides [88,89]. Natural peptides are derived from natural proteins, such as DNA-/RNA-binding proteins, viral particle envelope proteins, transactivators of gene transcription, plant circular skeletal proteins, and antimicrobial peptides. The proteins have been directly used as templates for CPPs development. These peptides show no cell specificity, and could deliver a wide range of substrates ranging from proteins, antigens, to PNAs into the cytoplasm and cell nucleus. Among these CPPs are Tat, vascular endothelial cadherin-derived CPP (pVEC), penetratin, which were truncated versions of full-length proteins. Later studies combined parts of several proteins to construct chimeric sequences such as transportan, Pep-1 or MPG with cell-penetrating properties [73,90] or rationally designed artificial CPPs, such as C105Y polyarginines, PepFects, MAP, or GALA, based on the structures of natural-derived CPPs [66,91,92,93,94]. Now purely synthetic sequences that can penetrate cells are available. More recent studies show that nonprimary and unnatural amino acids can further improve CPP properties. For example, resistance to cellular degradation can be increased by replacing lysine residues with ornithine [95].

Cell penetrating peptides are generally categorized based on their physical and chemical properties: cationic, amphipathic, and hydrophobic. Cationic CPPs are short amino acid sequences with a net positive charge at physiological pH, and they are mainly composed of basic amino acids (arginine and lysine) and histidine. Uptake studies with these peptides have shown that the number and order of the amino acids within the peptide sequence is critical for transport. They include peptides such as human immunodeficiency virus (HIV)-transactivating transcriptional activator (Tat), penetratin, and the polyarginine family. Amphipathic peptides have hydrophilic (usually cationic) and lipophilic regions that are responsible for forming the first contact and then facilitating translocation across the plasma membrane. Amphipathic peptides may be subcategorized as primary amphipathic, secondary amphipathic α-helical, β-sheet amphipathic, and proline-rich amphipathic CPPs. Examples of amphipathic CPPs include model amphipathic peptide MAP, MPG, BAC 7, CADY, pVEC, and Pep-1. In the third class are the hydrophobic peptides such as TP10, C105Y, Pep-7, and Bax-inhibiting peptide (BIP). They have a low net charge. Their hydrophobic motifs are crucial for internalization into the membrane core. The number of hydrophobic peptides is limited when compared to cationic, amphipathic peptides [66,79,84,88,96].

The entry mechanism of CPPs is dictated by their general physicochemical properties of the peptide, the nature of the cargo molecule, the linker between the peptide and the cargo, and the target membrane, and the experimental conditions [94]. The high heterogeneity present in this family of peptides hampers the identification of unique translocation mechanisms, as in the case of AMPs. Furthermore, a given CPP may utilize more than one pathway depending on the experimental conditions such as the peptide concentrations and incubation time [97,98,99,100]. Nevertheless, the two major routes for the entry of CPPs into the cells are endocytosis and direct penetration (or energy independent) [70]. 

The natural process of endocytosis occurs in two steps, endocytic entry followed by endosomal escape. The second step is critical in maintaining the activity of the cargo. Unfortunately, physicochemical properties of CPPs favorable for endosomal escape are not yet known. The different endocytic pathways used by CPPs include macropinocytosis, caveolae-mediated endocytosis, and clathrin-mediated endocytosis. Macropinocytosis is explained by the inward folding of the outer surface of the plasma membrane, resulting in the formation of vesicles called macropinosomes [100]. The latter two endocytic pathways are regarded as receptor-mediated endocytosis. Clathrin and caveolin proteins that cover the intracellular part of the membrane are involved in the mechanism of uptake. Due to experimental artifacts, the exact endocytic pathway that contributes to the uptake of CPPs is still unclear [94,99]. 

Direct penetration pathways used by CPPs include the inverted micelle [101], pore formation [102], carpet-like [103], and membrane thinning models [104]. The common initial step in all these pathways is the interaction of the positively charged CPP with the negatively charged components of the membranes to destabilize the structure of the membrane. This route is generally favorable at high peptide concentrations. Pore formation by CPPs can be explained by the barrel-stave and the toroidal models discussed above for AMPs. Pore formation is induced above a certain concentration threshold. The carpet-like and membrane thinning models are mechanistically similar to the nonspecific membrane permeabilization achieved by AMPs, as explained above. These models involve the carpeting and thinning of the membrane to enable subsequent translocation of the CPP as the concentration of the CPP gets above a threshold value.

## 4. Antimicrobial Peptides and Cell-Penetrating Peptides

The distinction between AMPs and CPPs is not always clear [105]. Indeed, many CPPs also serve as AMPs [106,107] and many AMPs have cell penetrating properties [7,29,108]. The presence of common characteristics, such as amphipathicity or harboring of arginine-rich regions, between the two groups also suggests that they can have dual roles. Sometimes, a single mutation can change the cell penetrating capability of a peptide toward antimicrobial activity or vice versa. For example, the arginine content in the CPP penetratin was found to influence its antimicrobial activity [107]. Similarly, increasing the cationic character of the CPP Pep-1 enhanced its antimicrobial activity [109]. Cationic antibacterial peptides (CAPs) have very similar physicochemical features with CPPs, yet they perform two different functions. Cell-penetrating peptides are known to penetrate eukaryotic cells without any apparent toxicity or damage while the main function of CAPs is to kill bacteria [110]. Interestingly, several CPPs have been shown to have antibacterial activity. Many CPPs, while not damaging eukaryotic cells, were found to be membranolytic in bacteria or in model membranes mimicking the bacterial bilayer composition [109]. The antimicrobial effect and specificity of the CPPs TP10 and pVEC were also investigated. Both CPPs are known to enter a range of bacteria and fungi. The uptake route involves rapid surface accumulation followed by cell entry. It was reported that TP10 and pVEC can enter both mammalian and microbial cells but they preferentially permeabilize and kill microbes [111]. The specific action of the peptides depends on the peptide sequence and structure, peptide concentration, membrane properties, and biophysical techniques to elucidate how the peptide behaves under different conditions. 

## 5. Characterization of Antimicrobial and Cell-Penetrating Peptides and Their Interactions with Membranes

Numerous biophysical techniques have been employed in an effort to examine the structural features of membrane active peptides found free in solution or as they insert themselves in the membranes. The secondary and tertiary structures, conformation, orientation, oligomerization states of the peptides in model membranes is of special interest because these peptides are membrane active peptides, some of them adopting a structure only upon binding to the membrane [112]. Thus, understanding the structural features of the peptides as they interact with the membrane is of paramount significance in understanding the mechanistic details of their insertion and membrane action. 

**Figure 5:**
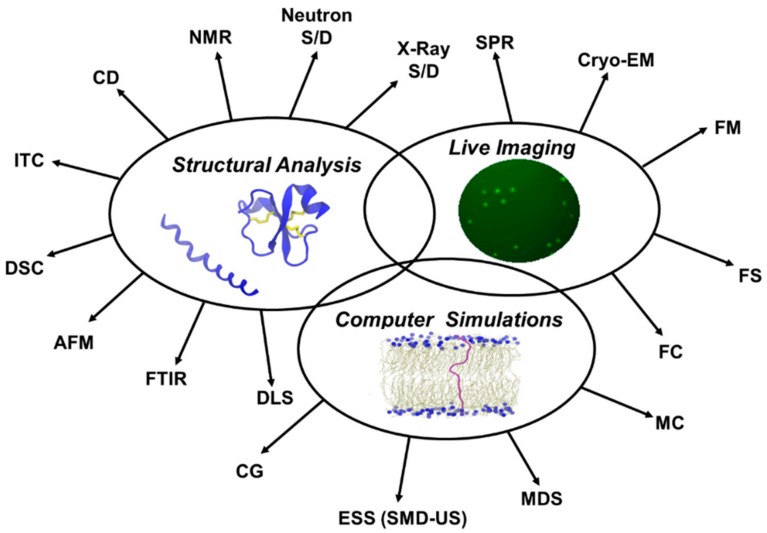
Major approaches used in biophysical characterization of membrane active peptides. X-ray S/D: X-ray scattering/diffraction; Neutron S/D: Neutron scattering/diffraction; NMR: Nuclear magnetic resonance spectroscopy; CD: Circular dichroism spectroscopy; ITC: Isothermal titration calorimetry; DSC: Differential scanning calorimetry; AFM: Atomic force microscopy; FTIR: Fourier transform infrared spectroscopy; DLS: Dynamic light scattering; CG: Coarse grained models; ESS: Enhanced sampling simulations (SMD-US): (Steered molecular dynamics simulations-Umbrella sampling); MDS: Molecular Dynamics simulations; MC: Monte Carlo calculations; FC: Flow cytometry; FS: Fluorescence spectroscopy; FM: Fluorescence microscopy; Cryo-EM: Cryoelectron microscopy; SPR: Surface plasmon resonance.

Different methods can be used to characterize the membrane active peptides with each method giving a different level of detail on the structure and mechanism of the peptide. Below is a summary of the commonly used biophysical techniques used, alone or in combinations, to study AMPs and CPPs and their interactions with biological membranes. Figure 5 is a schematic representation of the major biophysical characterization approaches discussed in this review. 

A brief list of these methods with examples of AMPs and CPPs is given in Table 1.

## 6. Structural Analysis

Biophysical methods are used to determine the secondary or tertiary structure of the peptide in the presence of the membrane, its orientation with respect to the membrane normal, and the thermodynamics related to binding in an effort to elucidate the mechanism of peptide uptake and membrane action. Currently, 313 AMP structures have been reported in the RCSB Protein Data Bank (PDB) [164] determined by solution NMR (234 peptides), by X-ray crystallography (73 peptides), by solid-state NMR (5 peptides), or by electron microscopy (EM) (1 peptide). The solid-state NMR structures are of piscidin and of protegrin-1 aligned in lipid bilayers [165,166]. The EM structure is that of a proline rich AMP in complex with the ribosome. On the other hand, the number of 3D structures of CPPs is limited, some examples are the solution NMR structures of penetratin [167], transportan [37], Tat [168], and crotamine [169], and the X-ray crystal structure of crotamine [170].

### 6.1. X-ray and Neutron Scattering/Diffraction

X-ray diffraction has been the primary method for the determination of the 3D structure of proteins. It is based on the diffraction of X-rays as they hit a surface, e.g., the protein crystal. The obtained diffraction data is then processed to resolve the structure of the molecule under study. While some AMP [34,171] and CPP [170] crystal structures [172,173] have been reported in the PDB [164], most peptides are small and flexible, and they resist crystallization. Furthermore, it is harder to determine the crystal structure of AMPs and CPPs in the presence of biological membranes [174], and there is only limited progress on the investigation of peptide–membrane interactions using X-ray diffraction methods [113,175,176,177,178,179,180]. 

The basic principle behind neutron diffraction is similar to X-ray diffraction, but it uses a beam of thermal or cold neutrons to obtain a diffraction pattern, which is then used to solve the structure without any radiation damage [181]. Exchange of H_2_O with D_2_O (deuterium oxide, heavy water) may provide information on the water content of peptide–membrane structure [182] and to investigate the water defects in the membranes during peptide translocation [183,184]. Neutrons with high-penetration depth are suitable to study structures with biological membranes [185], but the nuclear reactor required to produce neutrons is rather expensive.

### 6.2. Nuclear Magnetic Resonance Spectroscopy

Nuclear magnetic resonance spectroscopy is currently the prominent method in the analysis of membrane dynamics with peptides. It is based on observing the local magnetic fields produced by radio waves around atomic nuclei of a sample. The intramolecular magnetic field around an atom in a molecule changes the resonance frequency, thus giving access to details of the electronic structure of a molecule and its individual functional groups [186,187]. Most of the structures of AMPs and CPPs in the PDB have been determined by solution NMR spectroscopy. While solution NMR spectroscopy gives information on the solution structure of the peptides and their flexible regions, solid-state NMR spectroscopy gives information on the structures and orientations of membrane-associated peptides [188,189]. The low solubility of amphipathic or hydrophobic peptides hampers the use of aqueous buffers in solution-NMR. For this reason tetrafluoroethylene (TFE)/water mixtures or detergent micelles are commonly used as model systems to examine membrane proteins/peptides. The choice of the membrane model may influence the secondary structure of the peptide and the peptide may adopt a structure in the detergent different from the one in the lipid bilayer [36,190]. The choice of the mixtures may also affect the membrane curvature, and therefore the use of planar lipid bilayers could be more suitable [191,192,193,194]. Solid-state NMR spectroscopy gives high-resolution structures of peptides in disordered phospholipid bilayers as well as changes taking place in membranes after interaction with the peptides [195]. It provides information on the structure, dynamics, topology, and aggregation of peptides along with also the conformational and overall supramolecular characteristics of the lipid–peptide assemblies [196]. The most important features of solid-state NMR spectra are the anisotropic contributions of the chemical shift, the dipolar and the quadrupolar interactions [188]. The NMR active nuclei that are frequently used in the examination of lipid–peptide systems are phosphorous-31 (^31^P) and deuterium (^2^H). ^31^P is used to study the interaction between the peptide and the lipid head groups, whereas ^2^H-NMR is used to obtain information on the lipid tail dynamics and orientation [197,198,199,200]. The changes in static line, shape, width, and chemical shift anisotropy in ^31^P-NMR spectra can be used to investigate peptide–lipid interactions. The deformation of the membrane upon peptide entry can be detected by ^31^P, with the addition of the membrane disruptive peptide leading to signals detected at lower chemical shifts [201]. Proton decoupled ^15^N solid-state NMR spectroscopy can be used to study the alignment of the peptide with respect to the membrane plane [26,202].

### 6.3. Circular Dichroism Spectroscopy

Absorption spectroscopy, which uses the difference in the absorption between right-handed and left-handed circularly polarized light is known as circular dichroism spectroscopy. Circular dichroism (CD) can be used to study the secondary structure of peptides in solution since random coil, α-helix, and β-sheet have distinguishable CD spectra. While amphiphilic peptides form random coil structures in solution, they adopt α-helical or β-sheet structures upon binding to membranes [203]. Conventional CD gives information of a peptide in solution and the structural changes in a peptide upon binding [124,204]. The change in the structure upon binding the membrane can be used to estimate the ratio of bound to free peptides but traditional CD does not yield any information about the orientation of the peptide relative to the membrane. Thus, this approach is limited to samples in detergent micelles or liposomes or samples containing small sonicated lipid vesicles [112,204,205]. These studies try to mimic the bacterial outer leaflet, or lipopolysaccharide (LPS), to determine the structure of peptides in cell-like environments [124,196]. A variation of CD, oriented circular dichroism spectroscopy (OCD), is based on the Moffitt theory [206], which says one of the peptide transitions in a helix is polarized parallel to the helical axis. It is performed on macroscopically oriented samples to address the membrane alignment of a peptide in addition to its conformation. So far, it has been mainly used for the study of α-helical peptides [32,204,207,208]. Extensions of traditional CD spectroscopy such vibrational and electronic CD also exits as powerful tools for the study of membrane active peptides [209].

### 6.4. Calorimetry Methods

Isothermal titration calorimetry (ITC) is a powerful tool to characterize peptide–membrane interactions as well as secondary processes accompanying peptide–membrane interactions, such as membrane permeabilization, peptide induced lipid phase transitions, peptide aggregation at the membrane surface, and peptide conformational changes by detecting the total heat transferred during the ligand–protein binding event [210]. Using the assumption that binding heat is constant, the number of bound ligands can be determined from the total heat change. The binding constant and enthalpic and entropic changes of the binding process can be calculated with ITC [205,211]. An important advantage of ITC is that it can be performed with unmodified, native forms of molecules, hence it does not introduce artifacts [212,213]. Furthermore, ITC is a preferred approach for the analysis of CPPs that are coupled to larger biomolecules since CD and NMR spectra of these large molecules are very difficult to analyze [214] and unlike NMR techniques, ITC does not require large amounts of samples [215]. There are different applications of ITC to characterize the thermodynamic aspects of peptide–membrane interactions [211,216,217,218,219,220].

Differential scanning calorimetry (DSC) is valuable tool to get information regarding the level of interaction of peptides with particular membrane lipid components, that is, specificities of peptides for different target lipids [221,222]. Basically DSC monitors the changes in the phase transition temperature and thermodynamics of lipid phase transitions to get insight into the interaction mechanisms of peptides with lipids as the lipid membranes are reorganized or disrupted [223].

### 6.5. Atomic Force Microscopy 

Atomic force microscopy (AFM) is suitable to get information on the destabilization and restructuring of the membrane in the presence of peptides by resolving the molecular-scale topographical details at surfaces [224]. The AFM images give changes in cell height and surface roughness following treatment with a peptide. Unfortunately it is limited in the study of membrane mimic systems that do not provide sufficient topographical contrast, such as peptides that have inserted into a membrane [225]. Furthermore, since AFM identifies structures based on size and shape only, it lacks chemical specificity [210].

### 6.6. Fourier Transform Infrared Spectroscopy

The infrared spectrum of absorption and emission obtained by Fourier transform infrared (FTIR) spectroscopy is widely used for the study of the conformation and orientation of membrane-associated peptides and lipids [137]. The approach is based on vibration of atoms within molecules, which appear as peaks in an infrared spectrum. The spectral differences before and after perturbations recorded enable the detection of conformational changes. Fourier transform infrared spectroscopy allows the analysis of static and dynamic structure of peptides embedded in biological membranes, but pitfalls regarding to the absolute determination of secondary structure and orientation should be kept in mind [226,227].

### 6.7. Dynamic Light Scattering 

Dynamic light scattering (DLS), also known as photon correlation spectroscopy or quasi-elastic light scattering, is a label free method that measures the size and size distribution of particles in a suspension by primarily measuring the Brownian motion of the macromolecules. This motion depends on the size of the molecules, temperature, and solvent viscosity. In this technique, a monochromatic beam of light encounters a biomolecule solution and fluctuations in the scattered light intensity are analyzed [228,229,230,231]. Using this approach it is possible to get information on the microsecond timescale about the size distribution of aggregated species in the lipid bilayer [231,232]. For example, the antimicrobial activity of gomesin was related to its ability to induce vesicle aggregation, as detected by DLS [146]. Dynamic light scattering is a fast and noninvasive method that allows examination of the peptide–lipid system in its intact form but requires low particle concentration [233].

## 7. Live Imaging

The uptake of the peptide across the membrane can be followed using GUVs or liposomes [70,81,234]. These membrane mimics are often composed of mixture of predefined proportions of certain lipids while live cell membrane is composed of many different components in varying proportions with respect to time and space. Even though liposomes are very useful in identifying key uptake steps, the real question is whether a similar uptake is observed in live cells. For example, the stable pores observed with artificial membranes were found to have a transient nature when live bacteria were used. This highlights the complexity of cellular membranes compared to artificial membranes [235]. Advances in imaging techniques make it possible to monitor a peptide to obtain a spatiotemporal understanding of its interaction both with lipid mimics such as vesicles and live cells. Single cell imaging techniques allow a detailed description of the individual steps the peptide takes as it traverses or disrupts the membrane.

### 7.1. Surface Plasmon Resonance

Surface plasmon resonance (SPR) is a surface-sensitive technique, which relies on the surface plasmon resonance phenomenon. It is based on the real-time measurement of the change in adsorbed mass at the sensor surface as the peptides bind to selected biomimetic surfaces [236]. It allows the identification of the roles of different membrane components during the peptide–membrane interactions. Particularly, it can be used to get information on the kinetics of binding. Different studies have used SPR to study interactions of membrane active peptides with model membranes [237,238,239,240].

### 7.2. Cryoelectron Microscopy

Cryoelectron microscopy (Cryo-EM) has opened a new era within the structural biology community. The method is based on imaging frozen-hydrated specimens at cryogenic temperatures by electron microscopy to get 3D reconstructions of the macromolecules [241,242]. It minimizes the effects of high vacuum and radiation of other electron microscopy methods by using a biological sample in a frozen state after vitrification as it provides high resolution imaging [243,244,245]. Cryoelectron microscopy emerges as a potential alternative to X-ray crystallography because it requires much less sample and purity and does not require crystallization. Single-particle cryo-EM, cryo-electron tomography, and electron crystallography are sub-disciplines of cryo-EM and all have been used to analyze biological structures in different contexts. The most commonly used one is the single particle cryo-EM. In this analysis, the identical images of a protein complex in different orientations are combined to construct the 3D structures [243,246].

This technique has also found useful applications in the study of peptide–lipid interactions by enabling direct imaging of peptide–membrane systems in environments that closely resemble in vivo conditions [247,248,249]. With cryo-EM, it is possible to directly image peptides as they perturb the membranes to induce pores even under conditions where the structures cannot be individually resolved [159,250]. In the presence of a membrane thinning peptide, the induction of a new lamellar structure as the peptides accumulate between the membrane bilayers can be visualized. Furthermore, membrane curvature can be visually examined as vesicles, curved structures and undulations are induced in the presence of peptides [116]. A unique feature of cryo-EM is that the high-resolution structures obtained can give mechanistic details about the antimicrobial of peptides that have intracellular targets. For example, the cryo-EM structure of ribosome in complex with apidaecin, a proline rich peptide that targets the ribosome, suggests that the peptide terminates translation by arresting the ribosome at the stop codon [251]. Unfortunately, due to the rigidness of the contrast mechanisms, cryo-EM lacks the flexibility offered by other approaches such as solution NMR or X-ray crystallography [252].

### 7.3. Fluorescence Imaging: Microscopy and Spectroscopy

The principle behind live, highly sensitive fluorescence imaging is commonly based on linking a fluorophore to the peptides and/or the surrounding lipids under study and following the peptide–lipid interactions but labeling a peptide or a lipid molecule with a fluorophore may modify the behavior of the molecule [205]. Intrinsically fluorescent aromatic amino acids may also act as fluorophores.

Fluorescence imaging has a broad applicability with small sample requirements [210]. In spectroscopy, the output data is represented by an emission spectrum which is then related to estimate interaction dynamics [253,254], while in microscopy direct images are obtained [255]. In addition to mapping the position and the orientation of peptides in lipid bilayer systems, fluorescence imaging allows probing the interactions of peptides with living cells, the internalization mechanisms of peptides though lipid membranes, and the localization of the peptides inside the living cells with a kinetic study of the process [67,94,210,235,256]. Further, it can demonstrate the complexity of the dynamics of interactions, by enabling the comparison of the processes with living cells and artificial lipid systems [235].

Fluorescence correlation spectroscopy (FCS) and Förster resonance energy transfer (FRET) are two fluorescence spectroscopy applications. Binding isotherms of peptide–lipid systems can be measured by FRET using labeled peptide as the donor and labeled lipid as the receptor while FCS analysis provides information about the diffusion coefficients and rates of fluorophore transitions [205,257,258,259]. Specifically, with FRET it is possible to monitor the entry and then quantify the ratio of CPPs in the membranes [67].

One limitation of fluorescence methods is that a fluorescent probe that is attached to the peptide can affect the amphipathic properties, uptake mechanism, and kinetics as well as bilayer interactions of the peptide [260]. The choice of the fluorescent probe is therefore critical and must not be disruptive. Fluorescent probes can also be used to monitor leakage induced by membrane active peptides [81,234,261].

### 7.4. Flow Cytometry

Flow Cytometry (FC) is a laser-based technology that measures the fluorescence and optical characteristics of a single cell (or its organelles e.g., nuclei) as it flows in a suspension through a measuring device. Its principle is based on light scattering and fluorescence emission of the particle, with fluorescent features derived from antibodies or dyes, upon illumination with a series of laser beams. The detected signals are then processed for analysis [262,263,264,265]. Flow cytometry gives a statistically relevant and quantitative signal of fluorescence very quickly and allows sensitive detection. The technique provides both qualitative and quantitative information [163,262,264].

In the study of membrane active peptides, FC is a fast method that enables real-time-resolved study of bacterial membrane permeabilization and its kinetics. This information may then be used to retrieve information on the molecular mode of action, target, and specificity of the AMPs [161,266,267,268]. In addition to addressing the mechanism of cell entry, FC is an especially reliable and robust method in the investigation of CPP internalization and subsequent intracellular trafficking to quantify the fraction of internalized CPPs [163].

Imaging flow cytometry (IFC) combines the features of FC that enables objective statistical analysis of high cell counts and the features of fluorescent microscopy that provides high-resolution images of cells [269]. Different from FC, IFC allows analysis of cellular events on the basis of both fluorescent and morphological parameters upon interaction with the peptide [270,271].

## 8. Emerging Approaches

The difficulty of conventional methods in structural characterization of short transmembrane peptides has recently led to the development of the channel current method [272,273]. The approach uses a droplet contact method to form a stable planar bilayer lipid membrane (pBLM) through which current passes. By interpreting the current signals, it is possible to categorize peptides based on their possible translocation mechanisms such as barrel-stave, toroidal pore, and penetration models. Further analysis using three parameters, namely the current amplitude, the duration time, and the time between spike signals, gives information on the size of the defect in the membrane, duration of the defect formation, and event frequency of the penetration [272].

Biomimetic high-performance liquid chromatography (HPLC) offers an alternative method for estimating in vivo peptide stability, peptide lipophilicity, and peptide affinity for the phospholipid membranes. It is based on measuring biomimetic properties using chemically bonded protein and immobilized artificial membranes as stationary phases [274].

## 9. Computer Simulations

Computer simulations can provide atomic level resolution about the peptide–membrane interaction. This information is especially complementary to imaging experiments with single cells [275] in describing the mechanism. Molecular Dynamics (MD) simulations [276], Monte Carlo (MC) calculations [277,278], and umbrella sampling [279,280,281] have provided detailed biophysical characterization of the peptide–membrane system. Simulations are commonly used on peptide–lipid bilayer systems to examine the behavior of membrane active peptides as they orient at the water–bilayer interface [44] and during or upon penetration into the membrane [276]. In most all-atom simulations, the lipid bilayer is composed of a single lipid species such as palmitoyloleoyl phosphatidylethanolamine (POPE), to represent the inner bacterial membrane, or a few species, such as phosphatidylethanolamine:phosphatidylglycerol (POPE:POPG) (1:3), but in reality the cellular membrane is asymmetric, curved, and comprises other lipid species such as lipid A [282]. Although, it is a powerful tool, MD simulations require substantial computing resources to examine biologically relevant system sizes and timescales, especially hampered by the slow lateral diffusion of lipids [283]. In an effort to examine the membrane uptake mechanism, which takes place across complex lipid bilayers on the order of minutes, some approximations can be made. Coarse grained (CG) models of the membrane, such as the MARTINI force field [284], which represents lipid, water and peptide with beads corresponding to multiple atoms [285], or implicit solvent models, such as the implicit membrane model (IMM1) [286] can accelerate MD simulations such that longer timescales can be sampled. Furthermore, the increase in computational speed allows the examination of larger systems comprising complex bilayers with multiple lipid species such as cardiolipin [287] or cholesterol [281]. Some studies have also used membrane-mimetic micelles instead of large lipid bilayers to reveal key interactions in peptide–membrane systems [288].

Simulations can provide information on the peptide–lipid interactions that initiate and facilitate the peptide binding/insertion process. Atomistic simulations can identify electrostatic interactions between the anionic membrane groups and the cationic peptide positively residues that are involved in the initial peptide–membrane contact [289,290]. They can also provide valuable information on the mechanism of peptide–lipid interaction toward differentiating between different working models [41]. Simulations can also shed light on the mechanism of selectivity against bacterial or mammalian cell membranes, giving details on the type of interactions and weak contacts (e.g., hydrogen and van der Waals) that drive the process forward [291,292]. Computational approaches can further give an insight to the initial electrostatic interactions that govern the binding of lipopeptides to the hydrophobic core of the membrane [293]. The atomistic level of detail afforded by simulations opens the path toward the design of novel peptide analogs with increased efficacy [294].

Computational techniques are especially powerful for examining the conformation, orientation, and lipid interactions of the peptide in the membrane at atomic level detail. Different studies have shown that the peptides rapidly fold into predominantly helical configuration giving details on the positions of different residues [295,296]. These simulations further tell if the preferred orientation of the peptide is parallel or perpendicular to the membrane normal and if salt bridges are responsible for stabilizing this orientation [295,297]. Simulations can also be used to predict the tilt angle and the rotation angle of the peptide and results show good agreement with experimental solid-state NMR results [41,298]. Simulations are able to reveal many structural details previously inaccessible, such as the immersion depth of the peptide in the membrane and the packing of the dimerization interface [299]. 

The translocation of the peptide across the membrane usually takes place on the order of minutes [275]. Enhanced sampling methods such as steered molecular dynamics (SMD) simulations [300,301], replica exchange umbrella sampling [280,281], metadynamics [302] simulations can be used to move the peptide from one side of the membrane to the other. These methods are complementary to AFM experiments and permit comparison of simulated and observed force-extension profiles [303,304]. Furthermore, enhanced sampling methods allow the calculation of the potential of mean force for the reaction coordinate and provide key information about the underlying energy landscape for the transport process [280,305].

## 10. Biophysical Focus on Amphipathic Membrane Active Peptides

Amphipathic membrane active peptides have a cationic region, which is thought to facilitate initial contact with the membrane, and a hydrophobic region, which facilitates transport across the hydrophobic core [306,307,308]. The cationic region is often arginine rich while the hydrophobic region is leucine rich. In this section, we describe some recent biophysical findings that provide information on the contribution of these regions to membrane uptake.

Many CPPs, such as pVEC (LLIILRRRIRKQAHAHSK) [309,310] or Tat peptide (GRKKRRQRRRPPQ) [311], and AMPs, such as melittin (GIGAVLKVLTTGLPALISWIKRKRQQ) [16], have cationic regions. The uptake of the charged lysine or arginine residues across the lipid bilayer should be energetically unfavorable, but the prevalence of arginine rich peptides as membrane active peptides has suggested that arginine delivery across the membrane is a facile process [312]. The molecular basis and energetics of the translocation of arginine-rich CPPs through membranes are still not well-established [313,314]. Arginine rich peptides were initially reported to translocate by endocytosis or other energy dependent methods that do not involve movement of arginine through the lipid environment. Later, this was challenged by findings in which arginine has been shown to translocate across the bilayer by forming a water pore around the hydrophilic residues [289]. Furthermore, MD simulations have shown that arginine delivery through the pores involves formation of a water bubble around the arginine and that after penetration of the membrane by one arginine, the other arginines move via a piggy back mechanism [315,316]. Furthermore, free energy calculations by umbrella sampling simulations on octaarginine peptides showed that as peptide concentration increased, the surface area of the lipid bilayer expanded due to insertion of guanidinium ions into the lipid glycerol regions suggesting a cooperative kinetic mechanism that acts above a threshold adsorption concentration [313]. Solid-state NMR spectroscopy showed that Tat binds at the membrane–water interface in a random coil orientation, but the shortened guanidinium–phosphate distances suggested that Tat interacts with the distal leaflet of the membrane, and translocates across the membrane via some transient defects [317]. This may be facilitated by the snorkeling of arginine residues to the distal membrane leaflet [318,319,320] or by thinning of the membrane [180]. Transient pore formation was found to be involved in melittin transport by dynamic imaging techniques also [235]. Stabilization of the transient pore formed in the membrane by adsorption of an arginine rich peptide was recently shown by MD simulations and free energy calculations [313]. On the other hand, Huang and Garcia have shown that free energy barrier for translocation across a pore path is 80 kJ/mol lower than translocation along a pore free path [314]. These results suggest that transient water pores that form in the membrane and the water ‘bubble’ that forms around arginine during translocation facilitate delivery of arginine rich peptides. 

Leucine is another residue commonly found in membrane active peptides. Leucine is in fact the most abundant sidechain in all proteins [321] and its abundance has been linked to its ability to adopt multiple conformations [322]. In addition to its higher relative abundance, leucine rich regions commonly have specific roles as nuclear export signals [323,324], in DNA binding [325] or as neighbors to active sites [326]. Furthermore, transmembrane helices are enriched in leucine [327] and oligoleucines, but not oligoalanines, were shown to self-assemble in membranes [328]. The AMP database APD3 is also abundant in glycine, lysine, and leucine residues [5]. Leucine rich regions are commonly found in amphipathic CPPs such as pVEC (LLILL…) [309], transportan (…LLGKI…) [80], PepFect 14 (…LLGKLL…), which is derived from transportan [329,330], LAH4 (…LLALA…), and LL-37 (LLG…).

MacCallum and coworkers suggested that the cost of burying the charged arginine residue, found in many cationic peptides, can be offset by the favorable free energy associated with burying leucine [331]. Leucine was shown to partition favorably to both the interfacial region and the bilayer core and burying this nonpolar residue in the hydrocarbon core of the bilayer was favorable. It was later shown by Sec61 translocon insertion efficiency measurements, OCD and MD simulations, that polyleucine segments with more than 10 leucines readily insert into the membrane. Furthermore, when the leucine stretch is flanked by positively charged lysines, helices shorter than 10 leucines can insert into phospholipid bilayer [332]. Later, Ulmschneider et al. showed by MDS that for such a polyleucine helix, the equilibrium is between the surface bound and the transmembrane-inserted states and the water soluble state is not populated [333]. They also showed that this partitioning is insensitive to temperature and hence a zero-entropy process. Later, they repeated the simulations but by introducing an arginine residue at the center of the polyleucine segment [295]. Again, the peptide was either positioned at the bilayer interface or across the bilayer; 14 or more leucine residues were shown to be sufficient to offset the cost of transferring a single arginine. These results that show that leucine–arginine motifs have favorable interactions with the membrane are in agreement with the identification of a LRLLR conserved motif in spontaneous membrane translocating sequences [334]. Fuselier and Wimley showed that other variants of this motif also translocate across palmitoyl-oleoyl-phosphatidylcholine (POPC) vesicles and arginine placement within the LRLLR motif variants affected the concentration dependence of translocation [335]. Scanning electron microscopy (SEM) images of bacteria obtained in the presence of a chimeric peptide that combines a LLIILRR region with a β-lactamase inhibitor peptide showed that incubation with the chimeric peptide resulted in elongation of the cells suggesting that the peptide has an inhibitory effect on cell division and does not simply destroy the membrane. This gain of antimicrobial property with the addition of the Leu-rich region to an inhibitory peptide could be a powerful strategy in the design of novel AMPs [336].

In the following subsections of the current review, we review the advances and mechanistic details of two amphipathic peptides, melittin, an AMP and pVEC a CPP, obtained using different tools.

### 10.1. Melittin

Melittin, a 26 residue cationic amphipathic peptide with the sequence GIGAVLKVLTTGLPALISWIKRKRQQ, is the principal toxic component of bee venom [16] with antimicrobial, antiviral, antifungal, anticancer [337], and anti-inflammatory properties [338,339]. The melittin monomer can be divided into three regions. (1) Hydrophobic amino-terminal region. (2) Amphipathic midsection. (3) Cationic carboxy-terminal region [340]. The Leu zipper motif, with a Leu/Ile residue at every seventh position, contributes to its hemolytic activity [341,342].

The crystal structure (PDB ID: 2MLT) [171,340] and the solution NMR structure of melittin [343,344] have been resolved more than 30 years ago. The crystal structure of melittin in an aqueous environment is a tetramer held together by hydrophobic interactions and this tetrameric structure is important for its aqueous solubility despite the prevalence of apolar residues [340]. Each melittin monomer forms two helices interrupted by proline at position 14, described as a kinked rod. The relative orientation of the two helices are significant for biological activity and melittin with a D-Pro^14^ substitution loses its cytolytic activity [345]. A recent investigation of the melittin solution structure in aqueous and TFE-containing solutions showed that the solution mixture contained both cis and trans forms of the Leu13-Pro14 bond [346]. Working with micromolar melittin, the authors showed the transition from a disordered state in aqueous environment to a helical form in TFE. Circular dichroism spectroscopy analysis of single-D substitutions within melittin has indicated that this partly altered the secondary structure, essentially eliminating hemolysis with no significant effect on the antimicrobial action [347].

Based on its crystal structure, melittin has been suggested to dissociate into its monomers and then to lie parallel to the bilayer to penetrate shallowly in the apolar region of the membrane [340]. It induces the formation of pores, which is linked to its amphipathic helical character. Pore formation was related to the ‘wedge’ effect that increases the area of the outer leaflet causing an asymmetry in area with the inner leaflet thereby inducing pore formation. Unbiased all-atom long-timescale (4 µs) MD simulations showed that melittin binds strongly to the lipid bilayer in an unstructured configuration. The peptide rapidly folds into a predominantly helical configuration with the helix long-axis oriented perpendicular to the membrane normal [295].

It is well known that a critical peptide to lipid ratio is needed to induce stable pore formation. Melittin has been shown to induce transient pore formation at low concentrations [348], stable pores at high concentrations [349], and it can act as a detergent at higher concentrations. The longevity and stability of these pores have been unresolved issues [208]. Leakage assays either with synthetic membrane mimics or with live cells have provided valuable clues about the transience of these pores. Krauson et al. used large unilamellar vesicles (LUV) to develop a two-step assay in which they could follow membrane permeabilization (step one) and equilibrium peptide pore presence (step two) for melittin [350]. Their results showed that melittin formed equilibrium pores only at peptide:lipid ratios of 1:200 or higher. Electrochemical impedance spectroscopy results [351] and dynamic imaging of live cells [235] also showed that equilibrium pores do not form and pore formation is only transient. The dynamic nature of the cell membrane allows formation of transient pores that continually form and close and it is through these peptide induced transient pores that the membrane becomes permeable. Cryo-transmission electron microscopy (Cryo-TEM) experiments showed that as the melittin to phospholipid ratio increased from 1:200 to 1:25, LUVs started losing their well-defined shapes and eventually, at very high ratios, the presence of melittin ruptured LUVs, which then underwent fusion, yielding new large vesicles with ill-defined shapes [160]. Experiments with GUVs showed that melittin distributes to both sides of the membrane before formation of stable pores above a critical peptide:lipid ratio. Anomalous X-ray diffraction of the pores in peptide–lipid multilayers suggested that the two lipid layers bend and merge through a toroidal pore. Oriented circular dichroism spectroscopy showed that the fraction of peptides perpendicular to the membrane was insufficient to line the pore, ruling out the barrel stave model [208].

Two recent fluorescence microscopy approaches using live-imaging tried to explain the interaction between melittin and model membranes and the sequence of events in melittin transport. Nelson and Schwartz used total internal reflection fluorescence microscopy to examine the interaction by dynamic single molecule tracking [282]. The authors showed that using asymmetric bilayers provided information about the electrostatics of interaction between the cationic peptides and the anionic membrane. By tracking many individual molecules simultaneously as a function of time, a trajectory of the individual melittin molecules as well as statistics about residence time were obtained to show that in the presence of more negatively charged membranes, the cationic peptides resided on the membrane longer and their diffusion was slowed down. Yang et al. used single-cell fluorescence microscopy to describe the sequence of events as melittin crossed the outer and then the cytoplasmic membranes of live *Escherichia coli* (*E. coli*) cells [275]. Their analysis yielded a very detailed and complex mechanistic explanation for melittin translocation [352]. They divided the translocation into seven membrane related events, starting with permeabilization of the outer membrane, inward facing periplasmic bubble formation, cytoplasmic membrane permeabilization, resealing of the outer membrane, resealing of the cytoplasmic membrane and finally, 2–20 min later, repermeabilization of both membranes. Transient permeabilization sites of the outer and cytoplasmic membranes were located at curved membrane surfaces and the binding of melittin induced cell-shrinkage and generates curvature stress. Transient pore formation had previously been observed for vesicles [350] and phosphatidylcholine (PC) bilayers [351] but by following GFP leakage, Yang and coworkers were able to capture temporal and spatial information about pore formation in live *E. coli* cells.

The contribution of membrane curvature to peptide uptake has been recognized for melittin. Curved membrane surfaces such as the septal regions and endcaps are permeabilized, possibly due to the high concentration of anionic lipids at these surfaces [275]. Many proteins bind to positively curved membranes and membrane curvature sensing has been proposed to be due to three possible mechanisms, namely wedge formation by an amphipathic helix, scaffolding by protein oligomers, or protein crowding. Bending of the membrane may lead to packing defects and/or changes in lipid order [353]. Peptide binding can induce positive or negative membrane curvature [354]. Melittin preferentially adsorbs to the lipid interface via its cationic residues and its free energy is lowest when it is at the lipid bilayer interface. This interaction distorts the bilayer, with a so-called wedge effect, creating a curved surface and increased interfacial area, which is released with pore formation [208].

**Figure 6:**
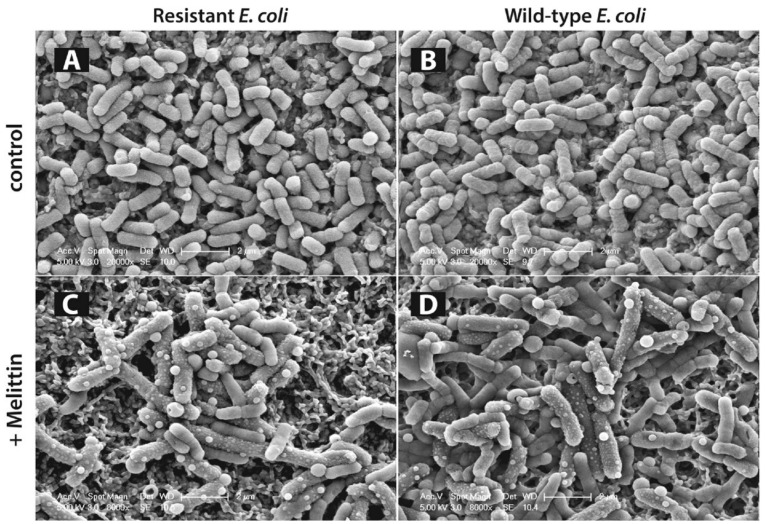
Scanning electron microscopy (SEM) imaging of antibiotic resistant and wild-type *Escherichia coli* (*E. coli*) cells on membrane filters. Antibiotic resistant *E. coli* cells in absence of any drugs (**A**), treated with minimum inhibitory concentration (MIC) of melittin (**C**), wild-type *E. coli* cells in absence of any drugs (**B**), treated with MIC of melittin (**D**) (reproduced with permission from a previous paper [336]).

We have previously imaged wild-type and ampicillin resistant *E. coli* cells under melittin treatment [336]. Scanning electron microscopy images have shown that in the presence of minimum inhibitory concentration of melittin, there was significant increase in the cell sizes, corrugation of the surfaces, and formation of abnormal textures similar to ‘membrane blebs’ over the cell surfaces. These images also showed that the antimicrobial effect of melittin was similar for both wild-type and ampicillin resistant bacteria (Figure 6).

A clearer picture of melittin membrane action emerges as imaging experiments [275,282] such as solid-state NMR spectroscopy [199] and MDS [46] provide more spatiotemporal resolution of the process. The amphipathic nature of melittin contributes to its interaction with the lipid headgroups via its cationic residues and its preference to assume a parallel orientation with the lipid bilayer [340]. With its positively charged residues extending toward the aqueous layer by snorkeling, the lipophilic region extends into the bilayer core [276]. Stretching of the long cationic chains is proposed to allow embedding deeper into the lipid region. Snorkeling to the opposite layer of the membrane then facilitates its transport across the bilayer after which melittin adsorbs in a parallel orientation on the bottom layer. The peptide distribution to both sides of the bilayer can then induce curving of both layers to merge and form a toroidal pore [275,286]. This picture is a short summary of the recent advances in elucidating melittin mechanism and is not complete because there are contradictory reports that support [286,355] or dispute [351] pore formation. However, some consistently observed phenomena point us toward understanding not only the melittin mechanism and but also the mechanism of other amphipathic peptides.

### 10.2. pVEC

pVEC is an 18 amino acid long CPP, derived from murine vascular endothelial cadherin (VE-cadherin) protein, which functions as the contact between adjacent cells by hemophilic dimerization. Originally reported as a CPP, it may also act as an AMP for different cells [279]. Similar to melittin, pVEC is an amphipathic peptide with the sequence LLIILRRRIRKQAHAHSK. The N terminus of pVEC is hydrophobic, with a LLIIL sequence, the middle part is cationic with four Arg and two Lys residues, and the C terminus is hydrophilic [310].

Circular dichroism spectroscopy analysis demonstrated that pVEC is found in its random coil conformation in pure water, containing a mixture of structural conformations and a low level of secondary structures [85]. Circular dichroism spectra obtained with small unilamellar vesicles (SUVs) of different phospholipids as well as in the presence of LUVs showed that pVEC adopts a β-structure in the presence of negatively charged phospholipids. However, it maintains its random coil conformation in the presence of neutral phospholipids. Furthermore, increasing the concentration of the phospholipids triggered conformational changes from random coil to β-structure. Overall, Eiríksdóttir et al. reported that pVEC lie within the sheet subgroup of CPPs that are characterized by a phospholipid-mediated conformational transition from a disordered state to a β-sheet structure [85]. In a recent study by Gong et al. with fungal cells, they also show that pVEC has a random coil structure in an aqueous environment [356]. Interestingly, contradictory to the finding by Eiríksdóttir et al. [85], they demonstrate by both live-cell CD and solution CD data that a hydrophobic environment drives the structural transition to an α-helix in pVEC and the that the membrane lipids are likely responsible for this transition. They propose that pVEC accesses the hydrophobic core of the membrane upon membrane insertion and assumes this α-helical conformation. Monte Carlo simulations further helped to see the shift in the conformation during penetration.

In vivo studies have shown that pVEC internalizes into several cell lines [309,310]. Furthermore, its uptake into gram-negative and gram-positive bacteria and fungi is also possible [356]. The N-terminal hydrophobic region (LLIIL) of this CPP was suggested to be crucial for this transport [304,309]. Structure–activity relationship (SAR) experiments showed a decrease in cellular uptake of pVEC by 50 to 75% upon substitution of the five N-terminal residues to L-alanine individually and complete loss of uptake upon deletion of three N-terminal hydrophobic residues or mutation of all five hydrophobic residues (LLIIL) to L-alanines in the N-terminus [309].

**Figure 7:**
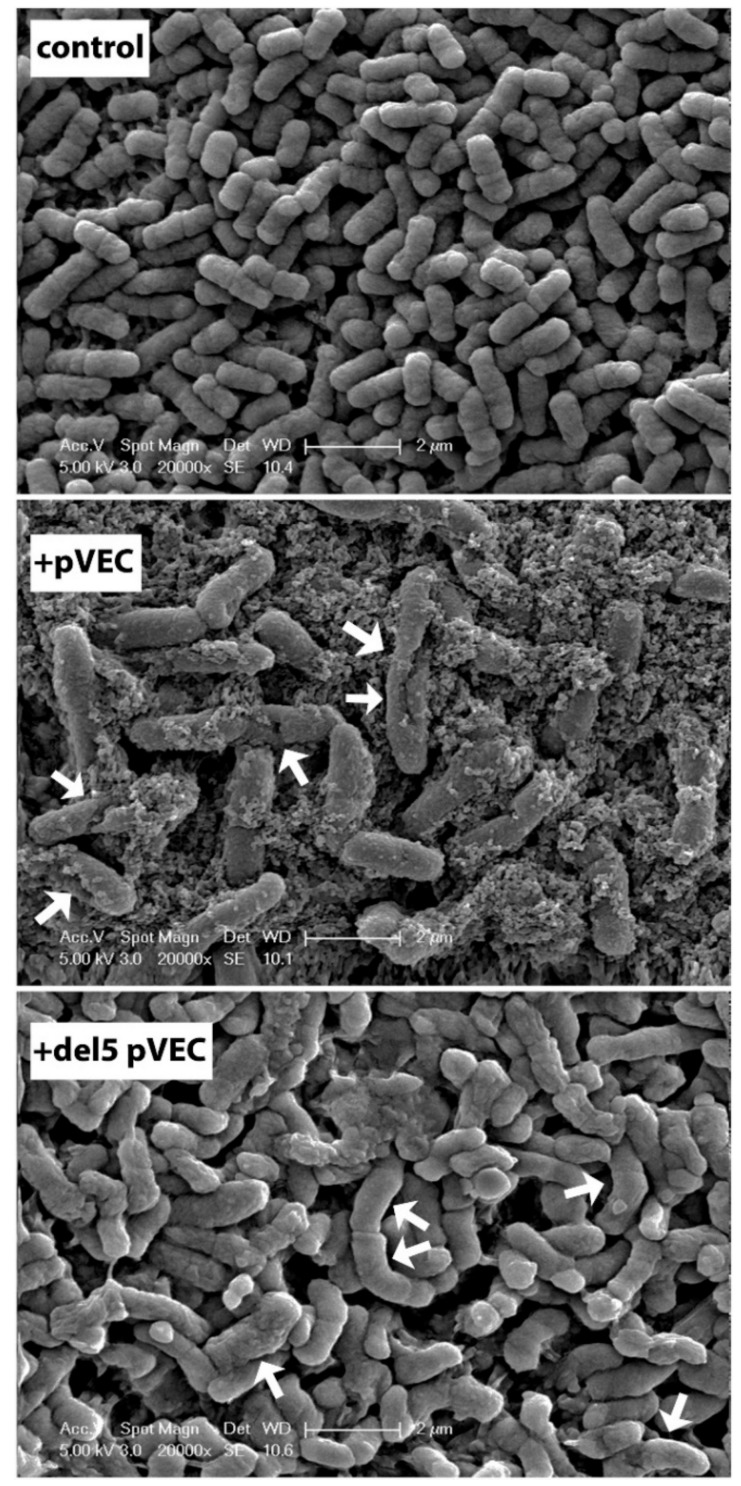
SEM imaging of *Escherichia coli* cells on membrane filters. Untreated *E. coli* cells (top), *E. coli* treated with MIC of pVEC (middle), and with 500 μM del5 pVEC peptide (bottom). Changes in surface structure due to peptide treatment are indicated by arrows (Reproduced with permission of the authors of a previous paper [279]).

Motivated by the information that the five hydrophobic N-terminal residues (LLIIL) of pVEC are critical for its transport, their importance on transport was further investigated by SEM imaging [279]. This analysis has shown that *E. coli* cells treated with around 50 μM pVEC had corrugated membrane surfaces with holes and membrane ruptures while incubation with much higher concentrations of (>500 μM) del5 pVEC (the five hydrophobic N-residues truncated) did not cause cell lysis or death and only membrane blebs were visible in the SEM images. These studies further underlined the importance of the N-terminal residues for transport (Figure 7).

**Figure 8:**
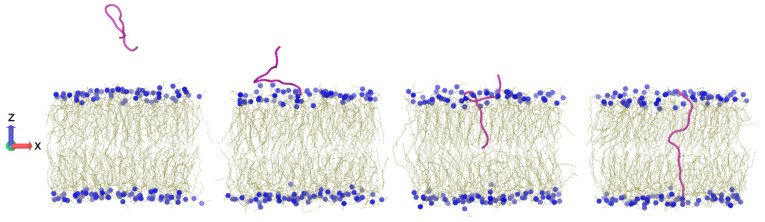
Translocation of pVEC across the lipid bilayer examined using SMD simulations [304].

In our work group, in order to elucidate the mechanism of pVEC uptake into the cell, we examined the translocation of pVEC, scramble pVEC (same residue content, mixed up sequence), and retro-pVEC (same residue order but in reverse direction) using SMD. In this approach, force is applied on a spring attached to one or more atoms of a system and then the center of mass of the selection is pulled in the specified direction [300]. We applied force on the peptide in an all-atom POPE bilayer–water system to move it from one side of the membrane mimic to the other (Figure 8). Our comparative results showed that the presence and precise order of the three parts of pVEC, namely the hydrophobic N-terminus, cationic midsection, and the hydrophilic C-terminus, is critical for the uptake of pVEC [304]. Later, we performed SMD and replica exchange umbrella sampling calculations to compare the free energy associated with pVEC and del5 pVEC transport across a POPE bilayer. Despite differences in uptake potential and antimicrobial properties of the two peptides, the calculated free energy values were similar, suggesting that pVEC uptake cannot be explained solely by passive transport of single peptides across the membrane. Indeed, the uptake of other peptides such as melittin [305], PGLa [327], Tat [289] has been shown to be concentration dependent.

Using CPPs for drug delivery into the cell has been a topic of interest. With the recent application of pVEC to cross the blood brain barrier [357], we believe that this CPP possesses significant potential for drug delivery applications.

## 11. Future Perspectives: Characterizing Membrane Active Peptides for Better Peptide-Based Drugs

Antimicrobial peptides that target essential bacterial machinery provide an important opportunity for the development of novel antimicrobial agents as bacteria evolve to acquire resistance against the currently available antimicrobial drugs [358,359]. On the other hand, CPPs have attracted attention not only because of their ability to carry and deliver cargo as Trojan horses, but also because of their use in imaging and visualization of intracellular event [360]. Despite the tremendous effort to understand the mechanism of action of membrane active peptides and to design new peptides based on these findings, the discovery and development of peptide based drugs are hampered by their limited membrane penetrability, stability, and bioavailability [361,362,363]. Most of the currently available peptide-based drugs target extracellular regions of receptors [364], while the number of AMPs and other peptide pharmaceuticals in clinical trials is increasing [365,366]. Modification with polyethylene glycol (PEG) [367,368] or nanoparticles [369], residue modifications such as mutation of lysine residues to ornithine [95], and cyclization of the peptides using internal linkages [370] are some of the approaches that have been used to enhance the stability and bioavailability of peptides. Targeted methodologies that pack the peptide in nanoparticles have been used to target only the cancer cells and hence reduce their toxicity related to their low selectivity [371,372]. For example, scorpion toxin, in which the main component is a 17mer peptide, has anticancer and antimicrobial properties due to its capability to form pores in biological membranes. However, it is not selective and therefore toxic. Misra et al. showed that packing this toxin in polymeric micelle nanoparticles that do not disintegrate in the blood stream can provide their safe delivery to cancer cells [373].

Peptide-based drugs draw attention because of their high specificity to their target and high activity compared to small molecule drugs [2]. Furthermore, with the cargo carrying properties of CPPs, they are finding use in the delivery of cancer drugs such as doxorubicin [260,374] and taxol family [375,376,377]. Using peptide conjugates for drug delivery into the cell has been a topic of interest and there are many anticancer [378], anti-inflammatory [379], and neurodegenerative [380] peptide conjugate drugs that are in clinical use or under investigation. Our group has also been focusing on combining the inhibitory properties of inhibitor peptides with CPPs [381]. Peptide vaccines (reviewed by Baig et al. [382]) have also gained interest in the immunotherapy of different types of cancer [383] such as breast cancer [384,385] or lung cancer [386], virally mediated diseases such as influenza [387], Human papilloma virus (HPV) [388,389], and HIV, and neurodegenerative diseases [382] such as Alzheimer’s [390]. These peptide vaccines are frequently delivered as emulsions [391] to facilitate uptake across the membrane. A telomerase derived peptide (GV1001) that combines anticancer properties [392,393] and cell penetrating capability [394] is gaining interest in the cancer immunotherapy [395]. There are 289 completed and 140 ongoing clinical trials [396] that include the keyword “peptide vaccine”. Crossing the blood brain barrier remains a challenge for the treatment of glioblastoma and other malignant brain tumors [397].

Curation of available AMP [5] and CPP [6] knowledge in databases opens the door to the design of novel peptide sequences [398] with antimicrobial activity [399] or cell penetrating capabilities [400]. As the myriad biophysical characterization methodologies ranging from structure analysis to high resolution live imaging to simulations provide more information on the mechanism of membrane action by these membrane active peptides, the rational design of novel peptide-based drugs will continue to attract intense research interest. The continuous development in imaging techniques as well as the increase in computational power opens new windows into the field and challenges some previous findings suggesting that, despite decades of research, there are still unresolved issues and the need for novel approaches in characterization continues.

## Figures and Tables

**Table 1:** Commonly used biophysical techniques for the study of membrane active peptides.

Method	Application	Advantages/Disadvantages	AMP/CPP	References
X-ray Diffraction/Scattering	3D Structure of the peptides Peptide–membrane interaction	Least expensive, high resolution, lower sample amounts, label-free. Large crystalline structures are needed, not suitable to study the structural dynamics of peptides in biological membranes, inability to image pores dynamically and in real-time.	Alamethicin Magainin 2 TAT Penetratin	[113] [114] [115] [116]
Neutron Diffraction/Scattering	3D Structure of the peptide Peptide induced pore formation	High penetration ability of neutrons, label-free. Nuclear reactor is required, expensive, large amount of samples.	Melittin Alamethicin TAT TP-2	[42] [117] [118] [119]
Nuclear magnetic resonance (NMR) spectroscopy	Structure of the peptides Orientation Membrane-peptide dynamics	High accuracy, label-free. Specialized expertise, expensive, need large amounts of peptides	Arenicin-2 Protegrin-2 Transportan 10 SAP	[120] [121] [32] [122]
Circular dichroism (CD) spectroscopy	Secondary structure of peptides and their changes in different environments	Quick estimation of secondary structure, cheap, simple Less accurate than NMR	Indolicidin Cecropin MPG CADY	[123] [124] [125] [126]
Isothermal titration calorimetry (ITC)	Thermodynamic properties of binding reaction Peptide induced lipid phase transitions Peptide aggregation at the membrane surface Peptide conformational changes	Complete and basic thermodynamic characterization, label-free Large sample amounts	Pardaxin PGLa riDOM r_9_ CPP	[127] [128] [129] [130]
Differential scanning calorimetry (DSC)	Changes in the phase transition temperature Thermodynamics of lipid phase transitions	Qualitative and quantitative measurement, provide information about physical and chemical change, label-free	Gramicidin KIGAKI MAP SAP	[131] [132]
Atomic force microscopy (AFM)	Structural changes on the membrane	Possibility of visualization of live cells in situ Do not provide sufficient topographical contrast, lacks chemical specificity, long image acquisition time, inability to image pores dynamically and in real-time	PGLa CM15 P_(α)_ TAT	[133] [134] [135] [136]
Fourier transform infrared (FTIR) spectroscopy	Conformation and orientation of membrane-associated peptides and lipids Analysis of static and dynamic structure of peptides embedded in biological membranes	Possibility of working in a wide range of environment, requires less time and sample, inexpensive compared to X-ray diffraction, NMR, CD spectroscopy Difficulties in determination secondary structure and orientation	Arenicin 2 Magainin 2 P_(α)_ Penetratin	[137] [138] [139] [140]
Surface plasmon resonance (SPR)	Real-time measurement of the change in adsorbed mass at the sensor surface as the peptides bind to selected biomimetic surfaces Kinetics of binding	High-surface sensitivity, real-time interactions analysis Expensive	Melittin Citropin 1.1 MCoTI-II Transportan	[141] [142] [143] [144]
Dynamic light scattering (DLS)	Measurement of size and size distribution of particles in a suspension Gives information about folding and conformation	Label-free, fast and noninvasive Needs low particle concentration	Maculatin Gomesin PepFect 3 (PF3) mtCPP-1	[145] [146] [147] [148]
Fluorescence microscopy	Visualizing the membrane interaction and intracellular distribution of fluorescent-labelled peptides on biomimetic membranes or live cells Effect of peptides on membrane integrity	Inexpensive, wide-range of applications	LL-37 Histatin 5 pVEC Vim-TBS.58-81	[149] [150] [151] [152]
Fluorescence spectroscopy	Kinetics of killing the live cell in real time Size and nature of transmembrane pores	Real-time interactions, quick, accurate Quenching of the contaminants	Cecropin A Trichogin GA IV TP-2 riDOM	[153] [154] [119] [129]
Förster resonance energy transfer (FRET)	Quantification of membrane disruption in live cells Determination of structural transition upon peptide–lipid interaction Peptide translocation across the bilayer	Highly sensitive Depend on concentration of fluorophores and distance between the donor and acceptor, require careful calibrations	PMAP-23 Halocidin 18-mer Transportan 10 Pep-1	[155] [156] [157] [158]
Cryoelectron microscopy (cryo-EM)	Determination of 3 D structure Visualization of peptide–membrane interactions	High resolution, no need for crystal structure, require less sample and purity Proper equipment and expertise	Magainin 2 Melittin Penetratin	[159] [160] [116]
Flow cytometry	Kinetics of permeabilization of live cells and cell killing Cell viability and metabolic activity Membrane structure and its integrity Membrane potential	Statistically relevant and quantitative signal of fluorescence, very quick, sensitive detection Expensive equipment	PepR Melittin Vim-TBS.58-81 Penetratin	[161] [162] [152] [163]

AMP: Antimicrobial peptide, CPP: Cell-penetrating peptide.
